# circEPSTI1 promotes tumor progression and cisplatin resistance via upregulating MSH2 in cervical cancer

**DOI:** 10.18632/aging.204152

**Published:** 2022-07-02

**Authors:** Peng Wu, Jing Qin, Lingyan Liu, Wupeng Tan, Linchen Lei, Jiayu Zhu

**Affiliations:** 1 Hengyang Maternal and Child Health Hospital, Hengyang 421001, Hunan Province, China; 2 Department of Pathology, The First People's Hospital of Changde City, Changde 415000, China; 3 Department of Obstetrics and Gynecology, Nanfang Hospital of Southern Medical University, Guangzhou, Guangdong, China

**Keywords:** circEPSTI1, circular RNAs, cisplatin resistance, MSH2, cervical cancer

## Abstract

CircRNAs (circRNAs) are a kind of non-coding RNAs which are extensively distributed in tissues. Previous investigations reported that circRNAs harbor indispensable roles in modulating the progress of multiple cancers. Nevertheless, the function along with the molecular mechanism of most circRNAs in cervical cancer progression was still not clear. Herein, we illustrated that circEPSTI1 is a remarkably upregulated circRNA, which we validated in tissues with cervical cancer along with cell lines. The biological role of circEPSTI1 in the advancement of cervical cancer was probed via loss-of function assessments. Silencing circEPSTI1 could diminish the proliferative capacity of the cervical cancer cells to spread. In cervical cancer cells, silencing circEPSTI1 dramatically elevated drug responsivity to cisplatin. Mechanically, RNA immuno-precipitation experiments and dual luciferase enzyme reporter experiments were conducted to reveal the molecular mechanism of circEPSTI1 in cervical cancer. In conclusion, this research premise identified the biological function of circEPSTI1-miR-370-3p-MSH2 axis in cervical cancer progression. Our result is significant for slowing the progress of and overcoming drug resistance of cervical cancer.

## INTRODUCTION

On the basis of global statistics, cervical cancer is the fourth cause of malignancy mortality of women all around the world [1]. In its early stages, surgery is the main treatment which may be combined with radiotherapy to strengthen the anti-tumor effect. Although treatment choices for recurrent cervical cancer are limited, chemoradiotherapy or chemotherapy only is by far the most suitable approach in late stages of cervical cancer [2]. Individuals with advanced cervical cancer or with recurring disease exhibit a dismal prognosis, with a one-year rate of survival of around 10% to 20% [3]. Chemotherapy is the conventional treatment form for those with advanced or recurring cervical cancer, and cisplatin tends to be one of the more successful options. Cisplatin is an adjuvant chemotherapy medicine for individuals with cervical cancer, although resistance to cisplatin is still an issue in treating these individuals [4]. As a result, novel techniques, and therapeutic targets for inhibiting cervical cancer growth and treatment resistance are pivotal.

CircRNAs (circRNAs) are a form of non-coding RNA found in a variety of species and have a covalent closed loop structure [5]. CircRNAs are ubiquitous in gene expression programs in human cells and are routinely synthesized in non-classical splicing forms [6]. CircRNAs have been documented to have a pivotal function in regulating gene expression post-transcriptionally [7]. CircRNAs bind to microRNAs (miRNAs) and block the dampening influence of miRNA on target mRNA, thus regulating the expression level of miRNA target key genes [8]. According to the previous investigations, circRNAs are the mediators of several diseases, including cancers [9]. One of the first studied circRNAs, ciRS-7/CDR1as, promotes the progress of multiple cancers and could serve as a prognostic biomarker in several malignancies [7, 10–15]. circRNAs have been proven to exert great influence on cancers for instance hepatocellular carcinoma [10, 16], breast cancer [17–19], bladder cancer [20, 21], and laryngeal squamous cell carcinoma [22]. Nonetheless, most circRNAs’ role along with the molecular mechanism in cervical cancer growth, remain unknown.

Herein, we discovered circEPSTI1 as an extremely upregulated circRNA via validating the cervical cancer tissues along with cell lines. Silencing of circEPSTI1 could attenuates the growth coupled with the spread ability of cervical cancer cells. Silencing of circEPSTI1 could remarkably elevate the drug responsivity to cisplatin in cervical cancer cells. RNA immunoprecipitation experiments and dual luciferase reporter experiments were conducted to reveal the molecular mechanism underlying involvement of circEPSTI1 in cervical cancer. Generally, this research identified the biological function of circEPSTI1- miR-370-3p-MSH2 axis in cervical cancer progression. Our result is significant for overcoming drug resistance in individuals with cervical cancer.

## RESULTS

### circEPSTI1 is upregulated in cervical cancer with circRNA features

**Figure 1:**
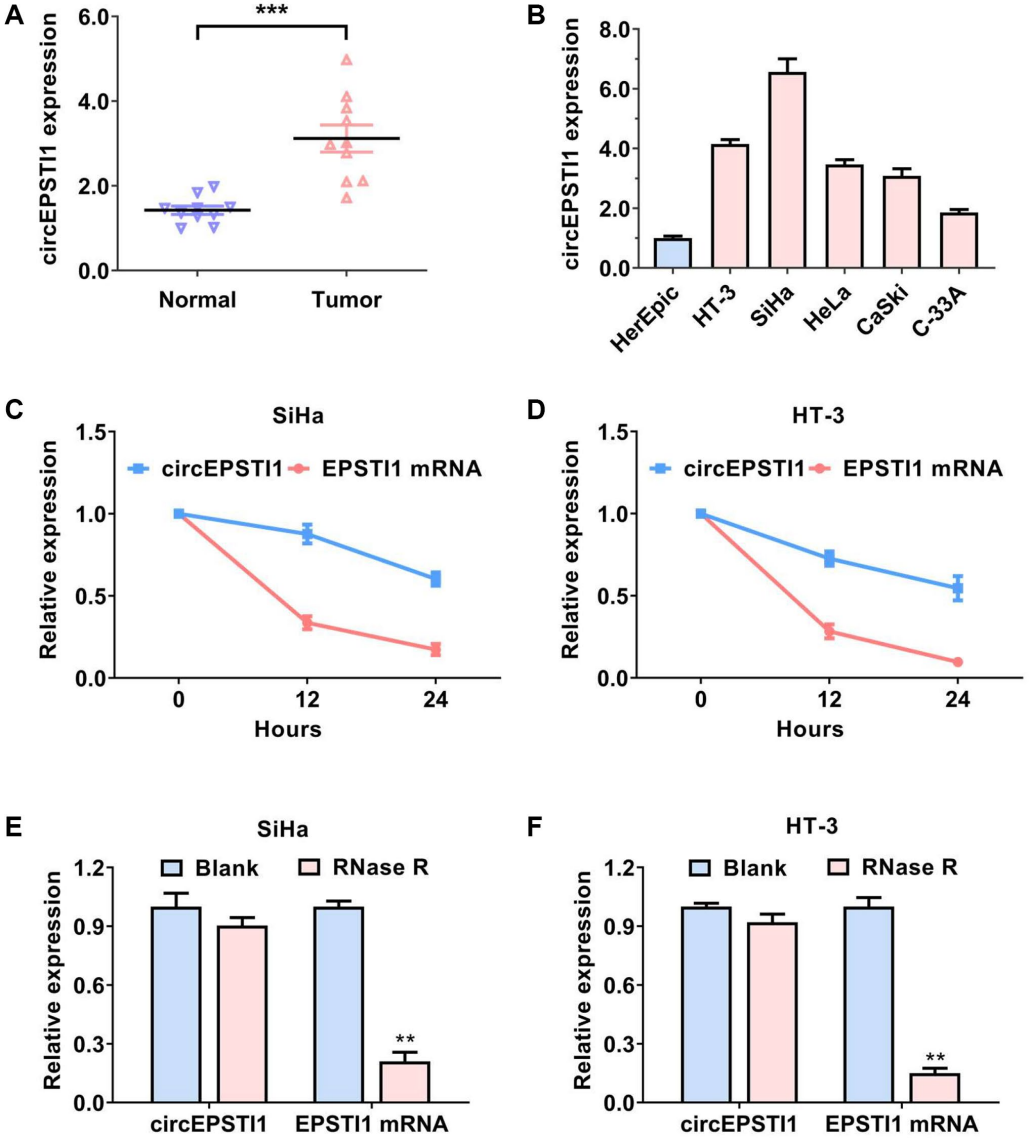
**circEPSTI1 is upregulated in cervical cancer with circRNA features.** (**A**) The contents of circEPSTI1 in ten paired cervical cancer samples and neighboring non-malignant tissues. (**B**) The relative contents of circEPSTI1 in normal HcerEpic cell line and cervical cancer cell lines. (**C**, **D**) Circular transcripts of circEPSTI1 were more stable, in contrast with that of linear EPSTI1 mRNA assessed via actinomycin D treated assay in SiHa and HT-3 cervical cancer cell lines. (**E**, **F**) The circular structure of circEPSTI1 was assessed via RNase R assay in SiHa, as well as HT-3 cell lines of cervical cancer. The analyzed gene was selected from the host genes of circEPSTI1 in cervical cancer tissues.

The content of circEPSTI1 was assessed in ten matched pairs of cervical cancer tissues along with adjacent non-malignant tissues. We established that circEPSTI1 was remarkably upregulated in the cervical cancer samples (Figure 1A). Then, the content of circEPSTI1 was examined in cervical cancer cell lines. circEPSTI1 content was elevated in cervical cancer cell lines, in contrast with that in the HcerEpic normal cell line (Figure 1B). RNase R assays along with actinomycin D assays were next performed to verify the circular characteristics of circEPSTI1 in cervical cancer. circKIF4A remained stable when exposed to continuously actinomycin D treatment (Figure 1C, 1D). As a circRNA, circKIF4A was also resistant to RNase R digestion in SiHa, as well as HT-3 cervical cancer cell lines (Figure 1E, 1F).

### Inhibition of circEPSTI1 attenuates the progression and cisplatin resistance of the cervical cancer cells

**Figure 2:**
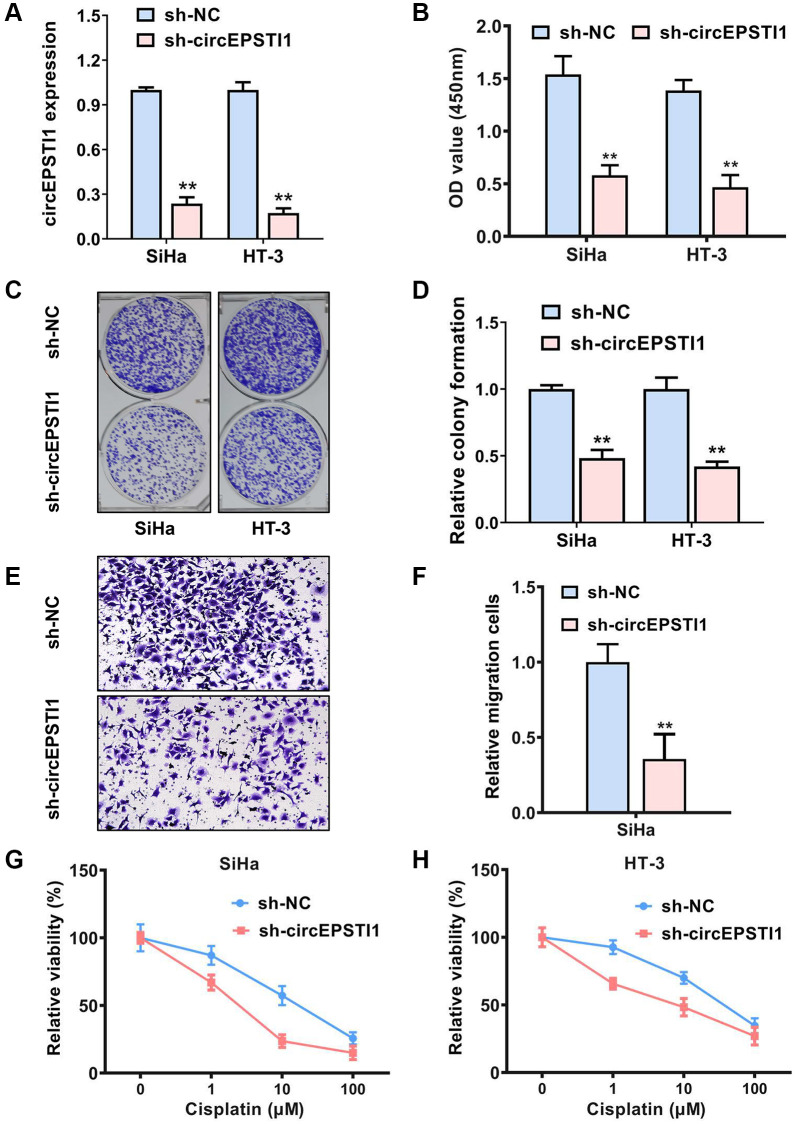
**Inhibition of circEPSTI1 attenuates the progress and cisplatin resistance of cervical cancer cells.** (**A**) The effect of shRNA knockdown of circEPSTI1 was assessed in SiHa, as well as HT-3 cell lines of cervical cancer, assessing via qRT-PCR. (**B**) CCK-8 assay was adopted to test the cell growth rate in SiHa along with HT-3 cell lines of cervical cancer. (**C**, **D**) Colony-formation experiments were conducted in SiHa, as well as HT-3 cell lines of cervical cancer. (**E**, **F**) Transwell assay for assessment of the metastasis potential of SiHa cervical cancer cell line. (**G**, **H**) Inhibition of circEPSTI1 remarkably increased the responsivity of cervical cancer to cisplatin treatment in SiHa along with HT-3 cervical cancer cell lines.

To explore the potential functions of circEPSTI1 in the progress and cisplatin resistance of cervical cancer, we conducted functional assays. Targeting the region of back-splicing junction of circEPSTI1, shRNA was designed to knockdown the expression of circEPSTI1 (Figure 2A). Revealed by CCK-8 assays, the rate of proliferation of the cervical cancer cells was remarkably reduced after silence of circEPSTI1 (Figure 2B). Knockdown of circEPSTI1 could also attenuates the colony-generation potential of cells in SiHa along with HT-3 cervical cancer cell lines (Figure 2C, 2D). Transwell assay was conducted to elucidate the migration potential of cervical cancer. We found that the amount of the migration cells was reduced after silencing circEPSTI1 (Figure 2E, 2F). Moreover, downregulation of circEPSTI1 could remarkably increase the drug sensitivity to cisplatin in SiHa along with HT-3 cervical cancer cell lines (Figure 2G, 2H).

### circEPSTI1 sponges miR-370-3p in cervical cancer

**Figure 3:**
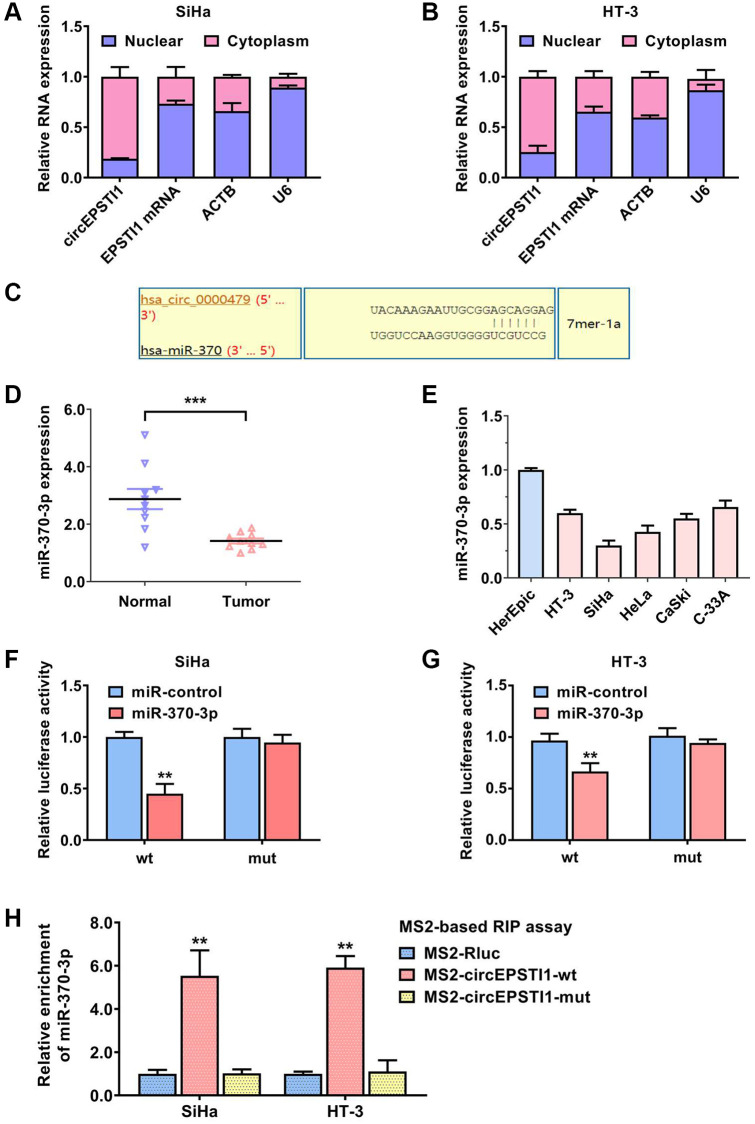
**circEPSTI1 sponges miR-370-3p in cervical cancer.** (**A**, **B**) U6, ACTB, circEPSTI1 and EPSTI1 mRNA contents in nuclear as well as cytoplasmic fractions were assessed via qRT-PCR assays in SiHa and HT-3 cell lines of cervical cancer. (**C**) Predicted docking sites of miR-370-3p within the circEPSTI1 sequence. (**D**) The contents of miR-370-3p in ten paired cervical cancer samples and vicinal non-malignant tissues. (**E**) The relative contents of miR-370-3p in non-malignant HcerEpic cell line and cervical cancer cell lines. (**F**, **G**) Dual luciferase reporting experiments revealed that the relative amount of fluorescence value was reduced after transfected with miR-370-3p in SiHa, as well as HT-3 cervical cancer cell lines. (**H**) MS2-based RNA immunoprecipitation assays were conducted to prove the docking of miR-370-3p on circEPSTI1 in SiHa along with HT-3 cell lines of cervical cancer.

To assess the potential molecular mechanism of circEPSTI1 in promoting cervical cancer progression and cisplatin resistance, we separated the nuclear and cytoplasm RNA and examined the abundance of circEPSTI1 in each proportion. circEPSTI1 was mainly existed in cytoplasm of SiHa, as well as HT-3 cervical cancer cells, detected by qRT-PCR analysis (Figure 3A, 3B). Calculated by the circinteractome algorithm, miR-370-3p was estimated to harbor the potential ability to cross talk with circEPSTI1 (Figure 3C). We collected ten cervical cancer specimens and their paired non-malignant tissues. miR-370-3p was remarkably down-regulated in cervical cancer tissues relative to normal tissues (Figure 3D). circEPSTI1 was also low expressed in cervical cancer cell lines in contrast with normal HcerEpic cell line (Figure 3E). Moreover, we carried out the dual luciferase reporting experiments to assess the direct interaction of circEPSTI1 and miR-370-3p. The data illustrated that the relative amount of fluorescence value was reduced after transfected with miR-370-3p in SiHa along with HT-3 cervical cancer cell lines (Figure 3F, 3G). MS2 mediated RNA immuno-precipitation assessments were also performed to evaluate the cross talk of circEPSTI1 and miR-370-3p in SiHa along with HT-3 cervical cancer cell lines (Figure 3G).

### MSH2 was the downstream target of miR-370-3p in cervical cancer

**Figure 4:**
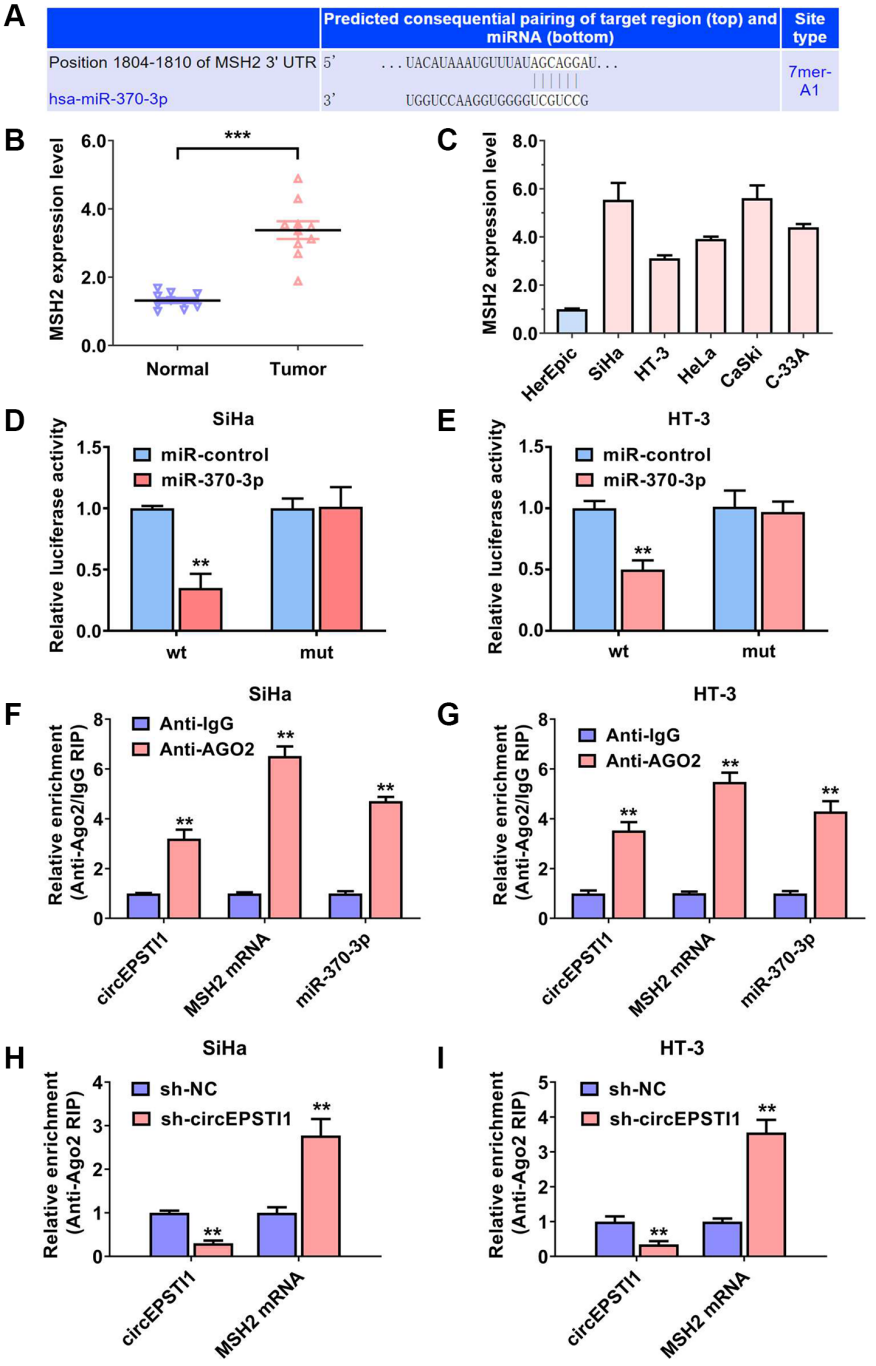
**MSH2 was the downstream target of miR-370-3p in cervical cancer.** (**A**) On the basis of the TargetScan data resource, MSH2 mRNA was the putative downstream target of miR-370-3p. (**B**) The contents of MSH2 mRNA in ten paired cervical cancer samples and vicinal non-malignant tissues. (**C**) The relative contents of MSH2 mRNA in normal HcerEpic cell line and cervical cancer cell lines. (**D**, **E**) Dual luciferase reporting experiments revealed that the relative amount of fluorescence value was reduced after transfected with miR-370-3p in SiHa, as well as HT-3 cervical cancer cell lines. (**F**, **G**) Abundance of circEPSTI1, MSH2 mRNA and miR-370-3p on the AGO2 target protein, assessed via RIP assays. (**H**, **I**) Enrichment of AGO2 protein to circEPSTI1 was diminished whilst MSH2 mRNA was increased after silencing of circEPSTI1 in SiHa along with HT-3 cervical cancer cell lines.

Next, we further assess the downstream of miR-355-5p in cervical cancer. According to the TargetScan algorithm, MSH2 was postulated as the potential target of miR-355-5p (Figure 4A). MSH2 encodes a protein with the function of DNA duplication and DNA repair, which facilitates the cell proliferation rate and chemotherapy resistance. The contents of MSH mRNA was detected in ten paired samples of cervical cancer tissues and the vicinal non-malignant tissues. Our data illustrated that MSH mRNA was remarkably upregulated in the cervical cancer samples (Figure 4B). Consistently, the contents of MSH mRNA was validated in cervical cancer cell lines. MSH mRNA was also high expressed in cervical cancer cell lines, in contrast with that in the HcerEpic normal cell line (Figure 4C). Luciferase enzyme reporter assays were carried out and the relative amount of fluorescence value was reduced after transfected with miR-370-3p in SiHa along with HT-3 cervical cancer cell lines (Figure 4D, 4E). AGO2 related RIP assays revealed that circEPSTI1, MSH mRNA, and miR-370-3p were all enriched in the AGO2 protein in SiHa and HT-3 cervical cancer cell lines (Figure 4F, 4G). Furthermore, the gather of MSH mRNA to RISC was reduced after knockdown of circEPSTI1 in both SiHa and HT-3 cervical cancer cell lines (Figure 4H, 4I).

### circEPSTI1 facilitates cervical cancer progression and cisplatin resistance through circEPSTI1-miR-370-3p-MSH2 axis

**Figure 5:**
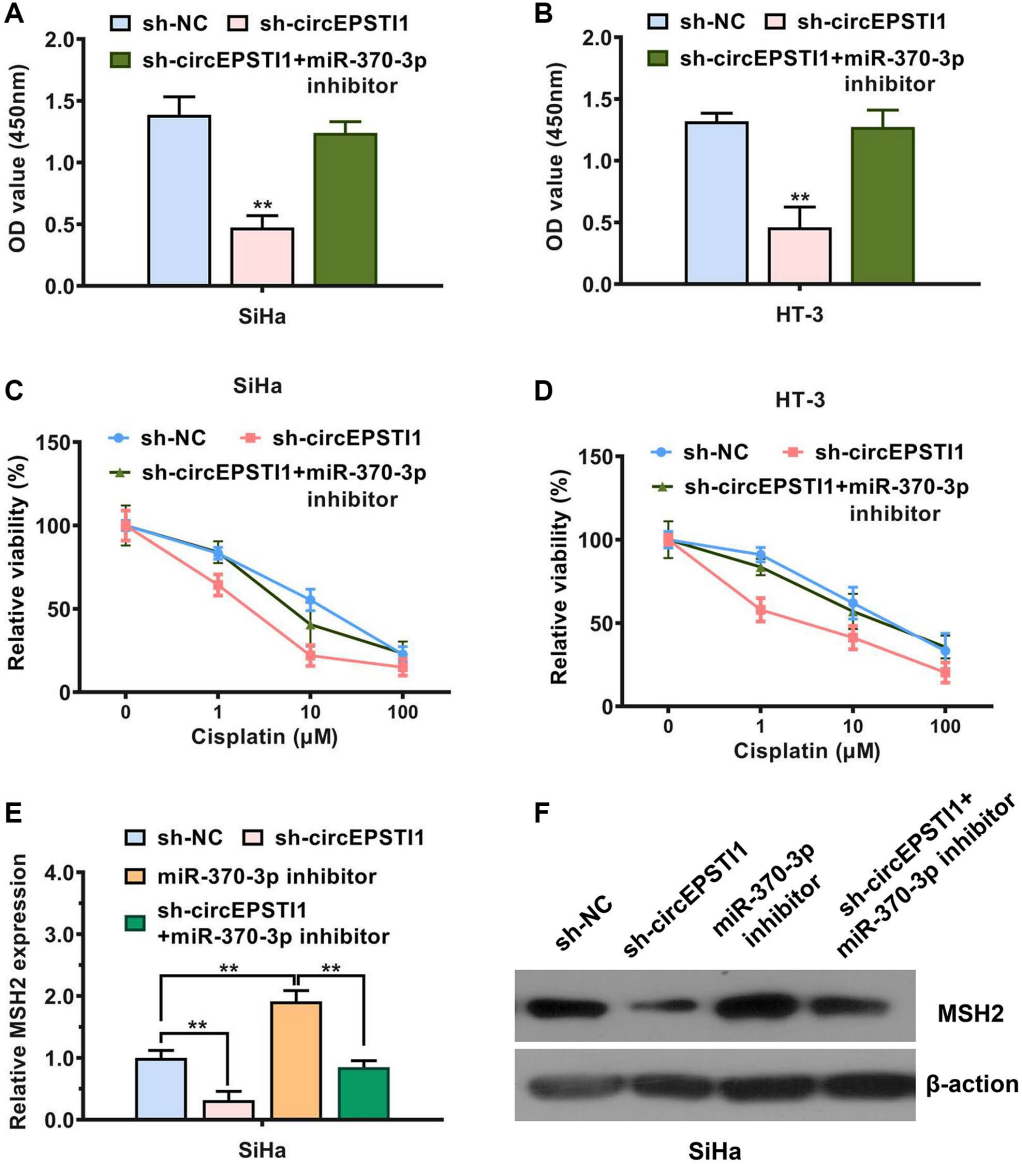
**circEPSTI1 facilitates cervical cancer progression and cisplatin resistance through circEPSTI1-miR-370-3p-MSH2 axis.** (**A**, **B**) CCK-8 assay for assessment of rate of cell proliferation in SiHa, as well as HT-3 cervical cancer cell lines. (**C**, **D**) Inhibition of circEPSTI1 remarkably increased the responsivity of cervical cancer to cisplatin treatment, whilst this effect could be rescues by miR-370-3p inhibitors in SiHa along with HT-3 cell lines of cervical cancer. (**E**) Expression of MSH2 mRNA transcript was reduced after circEPSTI1 inhibition and increased following the transfection with inhibitors of miR-370-3p, detected by qPCR analysis. (**F**) Knockdown of circEPSTI1 downregulated the MSH2 protein expression, which could be reversed through the miR-370-3p inhibitors in SiHa cervical cancer cells, illustrated by the western blot analysis.

To further examine the role of circEPSTI1-miR-370-3p-MSH2 axis in promoting cervical cancer progression and cisplatin resistance, we performed several rescue experiments. We found that the proliferation rate was significantly decreased by circEPSTI1 suppression, which could be reversed by the miR-370-3p inhibitors in SiHa and HT-3 cervical cancer cell lines (Figure 5A, 5B). Additionally, transfection of miR-370-3p inhibitors rescued the effect of circEPSTI1 knockdown on cisplatin resistance of SiHa and HT-3 cervical cancer cells (Figure 5C, 5D). Next, we detected the mRNA and protein expression level of MSH2 in each study group. Assessment via the qRT-PCR exhibited that the content of MSH2 mRNA transcript was upregulated by the introduction of miR-370-3p repressors (Figure 5E). Suppression of circEPSTI1 remarkably decreased the MSH2 protein expression, which could be reversed through the miR-370-3p inhibitors in SiHa cervical cancer cells, exhibited by the western blot analysis (Figure 5F).

## DISCUSSION

Overwhelming evidence illustrates that circRNAs have pivotal roles in the onset and progress of various disease, including cancer [23], dilated cardiomyopathy [24], heart failure [25], and unexplained recurrent spontaneous abortion [26]. CircRNAs can modulate the expression of many key genes via diverse mechanisms, consisting of cross talking with miRNAs, binding proteins, and encoding small molecule polypeptides [27]. Nevertheless, to date, few investigations have assessed the potential molecular mechanisms along with biological roles of circRNA in cervical cancer progression and cisplatin resistance.

Here, we identified circEPSTI1 as an upregulated circRNA in the cervical cancer tissues, as well as cell lines. Several loss-off function assays were conducted to figure out the biological role of circEPSTI1 in the progression of cervical cancer. Silencing of circEPSTI1 could attenuates the growth along with metastasis ability of cervical cancer cells. Silencing of circEPSTI1 could remarkably raise the drug responsivity to cisplatin in cervical cancer cells. RNA immuno-precipitation experiments along with the dual luciferase reporter experiments were utilized to reveal the molecular mechanism of circEPSTI1 in cervical cancer. DNA duplication and repair key gene MSH2 was regulated by circEPSTI1, through the mechanism of cross talking with miR-370-3p in cervical cancer cells.

According to endogenous RNA competition theory, matured mRNA, pseudogenes, lncRNAs, and circRNAs can regulate each other by sharing miRNAs through competing sponges [28, 29]. We illustrated that miR-370-3p was the mediator of the communication between circEPSTI1 and MSH2 mRNA. As tumor suppressor in multiple cancers, miR-370-3p has been proven as the upstream miRNA of several oncogenes. For example, miR-370-3p inhibits tumor growth and immune evasion by targeting CXCL12 axis in melanoma [30]. LINCRNA-00857 promotes lymphomagenesis and proliferation rate via miR-370-3p-CBX3 cascades in diffuse large B-cell lymphoma [31]. As the downstream target of miR-370-3p, MSH2 is important in DNA duplication and DNA repair in cancer cells. MSH2 lead to the resistance to platinum-based therapy via maintaining mismatch repair in muscle-infiltrative bladder cancer and cervical cancer [32, 33].

In conclusion, this research premise elucidated the biological function of circEPSTI1-miR-370-3p-MSH2 axis in cervical cancer progression. Our result is significant for overcoming drug resistance in patients with cervical cancer.

## MATERIALS AND METHODS

### Collection of clinical specimens

Fresh cervical cancer tissues along with vicinal non-malignant samples were acquired from Hengyang Maternal and Child Health Hospital and were put into liquid nitrogen to froze the samples at once. The Hengyang Maternal and Child Health Hospital’s Ethics Committee approved this research premise, which was conducted in conformity with the Declaration of Helsinki. Before taking part in this research, all cervical cancer subjects signed a written informed consent form.

### Cell culture

All cell lines utilized in this research premise including CaSki, HT-3, SiHa, HeLa, and HcerEpic were commercially provided by the ATCC library. Cell lines were inoculated following the supplier’s guidance and the instruction of the ATCC. DNA fingerprinting was adopted to verify the validity of all cell lines prior to the experiment. Routine detection of mycoplasma infection was also conducted.

### Western blot analysis

SDS lysis was adopted to isolate the total proteins, which was then introduced with PMSF to block degradation. After that, we fractionated the proteins in every study group and applied to PVDF membranes for 90 minutes at 300 mA. We inoculated the pr anti-MSH2 (1:1000, CST, USA) primary antibody at 4°C, followed by one hour of inoculation with the secondary antibody at RT (room temperature).

### qRT-PCR assay and analysis

TRIzol reagent (Invitrogen, United States) was applied to extract total cellular RNA. Afterwards, qRT-PCR were done using SYBR Premix Ex Taq Kit (Takara, Japan). The primers for circEPSTI1 are F: 5′-GGCAATTCAGAGAGAGAAGAGCA-3′, R: 5′-CCTGCTCCGCAATTCTTTGT-3′. The primers for EPSTI1 are F: 5′-ACCCGCAATAGAGTGGTGAAC-3′, R: 5′-GCTATCAAGGTGTATGCACTTGT-3′. The primers for ACTB are F: 5′-CATGTACGTTGCTATCCAGGC-3′, R: 5′-CTCCTTAATGTCACGCACGAT-3′.

### Actinomycin D resistance experiment

We inoculated the SiHa along with the HT-3 cervical cancer cell lines with 3 ug/ml actinomycin D for degradation of the linear mRNA transcription for different time (0-, 12-, and 24-hour). At the certain time point, the cells were all harvested and circRNA circEPSTI1 and the linear EPSTI1 mRNA were validated via qRT-PCR.

### RNase R digestion experiment

After isolating total RNA (2 ug) from SiHa cervical cancer cell line and inoculating for 30 minutes with RNase R (5 U/ug) or blank control at 37°C, the residual RNA was purified, then quantified via RT-qPCR.

### Cell counting kit-8 assay (CCK-8)

We re-suspended SiHa along with HT-3 cervical cancer cell lines, and 5000 sh-circEPSTI1 cells/well and 5000 sh-NC cancer cells/well were put into a 96-well plate, then left them to grow for three days at 37°C. Afterwards, 10 μl CCK-8 solution was introduced to every well of the 96-plate and allowed to grow for two hours. The absorbance of the senser at 450 nm in each well was measured using a microplate reader.

### Transwell assay

Firstly, 2.5 × 10^4^ SiHa cervical cancer cells were re-suspended and added to the upper chambers (withdrawn of FBS) and medium (with 20% FBS) was introduced to the lower chambers. The cells in the upper chambers were removed by a swab. The migrated cervical cancer cells were then imaged by a microscope (Nikon), after fixing and then staining with crystal violet (2.5%).

### Luciferase reporter assay

Dual-luciferase enzyme reporter experiment system kit (Promega) was used to assess the relative activity of the luciferase enzyme. SiHa along with HT-3 cervical cancer cell lines were inoculated into 3000 cells per well (96-well plate). The postulated miR-370-3p binding sites of circEPSTI1, and 3′-UTR of MSH2 mRNA was manually mutated. 48 hours before evaluation, established reporting plasmids (circEPSTI1-wt/mut or MSH2 3′-UTR-wt/mut) and the miRNA miR-370-3p mimics were co-transfected into cells for further test.

### RNA immunoprecipitation (RIP)

An anti-AGO2 antibody was adopted to perform the RIP assays for AGO2 protein. The relative RNA expression and abundance of circEPSTI1, MSH2 mRNA, and miR-370-3p was examined after purification of the obtained RNA. SiHa, as well as HT-3 cervical cancer cell lines were transfected with MS2-circEPSTI1 overexpression plasmid, MS2-circEPSTI1-mt overexpression plasmid, and MS2-Rluc overexpression plasmid. RIP assay was carried out after the incubation of cells for 48 hours. Following the purification of elution buffer of RNA complexes, the abundance of the miR-370-3p was determined.

### Statistical analysis

All statistical analyses were implemented in the SPSS 22.0 software (SPSS, USA). All data are given as the mean ± standard deviation (SD). Paired *t* test was used to compare the expression difference between normal and cervical cancer tissues. Student’s *t* test was adopted to compare the difference between two groups, with *P* < 0.05 signifying statistical significance.
